# The continued importance of animals in biomedical research

**DOI:** 10.1038/s41684-024-01458-4

**Published:** 2024-10-14

**Authors:** Michael C. J. Chang, Franziska B. Grieder

**Affiliations:** Office of Research Infrastructure Programs, Division of Program Coordination, Planning, and Strategic Initiatives, Office of the Director, National Institutes of Health, Bethesda, Maryland USA

**Keywords:** Experimental models of disease, Medical research

## Abstract

In the rapidly evolving field of biomedical research, the role of animal models has long been a topic of scientific and ethical debate. However, despite advancements in alternative modeling approaches, animal models remain an essential component of scientific discovery and medical advancement.

Animal models have been the cornerstone of biomedical research for decades. They provide invaluable insights into human disease mechanisms, preventative and therapeutic targets, and treatment strategies, making them indispensable tools for translational research. Each type of animal model offers distinct advantages and insights. The diversity among existing animal models allows researchers to select the most appropriate system for studying specific diseases, therapeutic interventions and biological processes, ultimately enhancing the relevance and applicability of the findings to human health. Characterizing, validating and selecting the right model is crucial for every area of scientific research. Whether investigating pathology, physiology, pharmacology or biochemistry, specialists and experts are needed to identify the most appropriate model to address specific research questions aimed at improving health outcomes. All investigators and studies that are using an animal model to explore biomedical questions across the disease spectrum must rigorously assess the model’s accuracy to confirm that it faithfully replicates the condition or disease that is under investigation.

When evaluating therapeutic or other interventional strategies, it is essential to ensure that the chosen model is suitable for the intended intervention. This includes considering whether the model’s anatomy, metabolism and environmental conditions are compatible with the proposed treatment strategy and whether they allow for scientifically sound evaluation of the outcomes. Additionally, the genetic diversity and manipulability of animal models have a critical role in study planning. Defining the genetic background of the model during the selection phase is critical, especially when using knock-out or knock-in mutations that create loss-of-function variants because these can produce phenotypes that accurately mimic disease surrogates.

Examples of animal models used in biomedical research include rodent models and nonhuman primate models. Rodent models have been extensively employed for genetic studies and disease modeling, particularly in research on cancer and neurological disorders. Rodents’ genetic similarity to humans, coupled with the availability of transgenic rodent models, makes them invaluable in these fields. In addition, the extensive array of defined markers and tools – such as monoclonal antibodies, cloned or natural proteins and specialized cells – further enhances their utility, enabling precise and robust investigations into complex human diseases. For example, unlike *in vitro* models or nonmammalian species, Syrian hamsters (*Mesocricetus auratus*) exhibit a disease progression that mirrors the clinical and pathological features of COVID-19 in humans, including viral replication followed by lung inflammation in the respiratory tract^1,2^. Their ability to replicate human-like disease symptoms, transmission and immune responses makes them invaluable for understanding SARS-CoV-2 infection and developing effective interventions. Likewise, humanized mouse models have become increasingly important in biomedical research because they can express human genes or harbor human tissues, making them better suited for studying diseases that have specific human pathophysiological characteristics, such as cancer, infectious diseases and autoimmune disorders^3–5^. Nonhuman primate models have a crucial role in biomedical research due to their close genetic and physiological similarities to humans. This characteristic makes them invaluable for studying complex diseases that cannot be fully replicated in simpler models, such as neurodegenerative disorders and infectious diseases^6^. The intricate interplay between genetics, environment and disease in primates allows for a more accurate representation of human conditions. This is especially important in preclinical studies on vaccine and therapy development, where understanding the systemic effects of treatments is essential.

Ethical considerations around animal research are of paramount importance and are addressed through rigorous regulations and ethical standards. Protocols that establish standards for treatment of animals in research settings ensure that animals are treated with care throughout their lives, and maintaining scientific validity is essential because stress and inadequate care can affect research outcomes. Regulatory frameworks – including the Animal Welfare Act (AWA), the only federal law in the United States that regulates the treatment of animals in research, and the Public Health Service (PHS) Policy^7^, which ensures compliance with AWA and other relevant regulations – provide guidelines for the proper care and treatment of animals in PHS-funded research. In addition, the *Guide for the Care and Use of Laboratory Animals*, published by the National Research Council of the National Academies, sets standards for the care and use of laboratory animals, including housing, nutrition and veterinary care. Institutional Animal Care and Use Committees (IACUCs) are required at every research institution to oversee and evaluate animal care and use programs, including reviewing research proposals to ensure ethical standards are met. NIH supports these efforts through training and education programs and other resources that ensure researchers and staff understand and implement ethical practices in animal research. Furthermore, NIH offers funding and resources for research aimed at improving animal welfare and developing alternatives to animal use.

Biomedical researchers are committed to the principles of the 3Rs – replacement, reduction, and refinement – and have always relied on a combination of innovative methods, models and technologies to answer complex questions about human health and disease. NIH has exemplified this commitment by promoting the integration of animal and non-animal methods to optimize research outcomes while minimizing animal use. Although animal research remains a crucial resource for understanding the complexity of human biology, rapid technological advancements are driving the development and adoption of complementary non-animal-based approaches. Among these novel alternative methods is human tissue-on-a-chip technology, also known as organs-on-chips, which incorporates engineered or natural miniature tissues cultivated within microfluidic chips. These chips are designed to simulate human physiology by controlling cell microenvironments and preserving tissue-specific functions. By integrating advances in tissue engineering and microfabrication, organ-on-a-chip technology has attracted attention as a next-generation experimental platform for drug development, disease modeling and personalized medicine. Researchers have used these chips to create models of human lung tissue, for example. A sophisticated microfluidic bronchial-airway-on-a-chip, lined with highly differentiated human bronchial airway epithelium and pulmonary endothelium, was developed and utilized to simulate viral infection^8^. This model demonstrated strain-dependent virulence, cytokine production and recruitment of circulating immune cells, providing critical insights into the pathophysiology of respiratory infections. It was used to screen drugs for use against influenza and SARS-CoV-2. Although organ-on-a-chip technology offers numerous advantages, it has several weaknesses and limitations, including the complexity of the biological systems being studied, lack of systemic interactions and limited long-term studies, as well as microenvironment limitations. Therefore, although organ-on-a-chip models can provide valuable insights, they should be considered complementary models to animal research. Despite the promise of such *in vitro* devices, major challenges remain when the research goal is disease prevention and treatment. To elicit a protective immune response against pathogens like viruses, it is essential to use an organism with a fully functional immune system. Although isolated immune cells can provide preliminary insights into which arm of the immune system may be activated for protection, testing the effects of pathogen or toxin exposure requires a whole-body approach. Specifically, it is the coordinated interaction between immune cells and the affected organs or systems that ultimately determines the success of disease protection. Similarly, critical questions regarding vertical and horizontal transmission of infectious pathogens cannot be adequately addressed in a cell system, even though mathematical models might offer some insights into demographic spread. For instance, the question of whether a mother can transmit an agent to her unborn or nursing child is best answered using an animal model that accurately replicates the disease in humans. Furthermore, evaluating the influence of environmental factors on disease outcomes poses major challenges *in vitro* or *in chemico*. Confounding factors such as environmental toxins, nutritional imbalances, smoke exposure or extreme environmental temperatures can profoundly affect disease progression. Currently, none of the alternative or complementary models available can reliably assess disease progression under these complex conditions. Although novel complementary models offer promising opportunities to advance drug development and precision medicine, they are just that — complementary. Animal models continue to provide valuable insights into complex whole-body interactions.

Building on the human organs-on-chips technology and other alternative methods, such as computational simulations, NIH has announced a new program, Complement Animal Research In Experimentation (Complement-ARIE). The Complement-ARIE program aims to speed the development of alternative models in biomedical research that complement traditional animal studies. The program is dedicated to the development and integration of cutting-edge *in vitro*, *in silico* and *in chemico* methods, with the goal of markedly reducing dependence on animal models. By supporting research that employs these innovative methods, Complement-ARIE aims to enhance the precision and ethical standards of experiments and deepen our understanding of complex biological processes. Despite the program’s aim to complement animal research with alternative methods, it may not substantially reduce the overall use of animals in studies. Some studies may still rely heavily on animal models due to the limitations of current alternative technologies. Additionally, successfully integrating animal research with these complementary methods will be challenging^9^ and require considerable collaboration and coordination among researchers with different expertise. These challenges encompass the need for seamless cross-disciplinary communication and meticulous coordination in experimental design^10^. Integrating animal research with complementary methods demands careful planning to ensure that studies are not only comparable but also synergistic. Researchers must align their objectives, methodologies and timelines to bridge the gaps between different disciplines, ensuring that the combined efforts yield robust and cohesive results. Moreover, integrating data from diverse sources – such as animal models, *in vitro* systems and *in silico* simulations – presents considerable challenges. The complexity of data integration and interpretation arises from the inherent differences in data types, scales and resolutions across these platforms^10^. Successfully merging these distinct data sets requires sophisticated methodologies and a deep understanding of each system’s unique contributions to ensure that the combined data provides meaningful and accurate insights. Differences in the types and scales of data generated may complicate the synthesis of research findings and results. Despite these concerns, Complement-ARIE exemplifies this commitment by promoting the integration of animal and non-animal methods to optimize research outcomes while reducing animal use. The program highlights NIH’s strong commitment to refining research methodologies and broadening the range of tools available for scientific exploration.

Although advancements in alternative methods will revolutionize biomedical research, the continued use of animal models and support of their resource infrastructure remain crucial. Animal models provide a comprehensive, whole-organism perspective that alternative methods cannot yet fully replicate. They deliver critical insights into systemic interactions, long-term effects and multifactorial responses to treatments, which are essential for understanding complex diseases and developing effective therapies. Furthermore, animal models are essential in the regulatory approval process because they are used to ensure the safety and efficacy of new drugs and medical devices before progressing to human clinical trials. The COVID-19 pandemic underscored the irreplaceable role of animal models, especially nonhuman primates, in the rapid development of vaccines. By the end of 2021, the U.S. had recorded over 103 million COVID-19 cases and 1.19 million deaths, while vaccines were estimated to have prevented 1.1 million additional deaths and over 10 million hospitalizations^11^. The COVID-19 pandemic served as a reminder of the reality that without nonhuman primates and other animal models, it is likely that we would not have had vaccines and effective treatments to combat the pandemic. The pandemic also highlighted the necessity of well-equipped research infrastructure, such as the biosafety level facilities that are crucial for safely handling highly infectious pathogens. Training researchers, including veterinary scientists, to operate in these high-containment environments was equally important for advancing research swiftly and securely. This synergy of robust animal models, advanced infrastructure and skilled researchers was key to the expedited development and approval of COVID-19 vaccines, demonstrating the critical need to maintain and support these resources for future health challenges. By investing in infrastructure for animal research, we ensure our capacity to quickly respond to emerging health threats while upholding high ethical standards and advancing animal welfare. This comprehensive approach, in conjunction with emerging alternative platforms, bridges the gap between basic research and clinical application, driving innovations that enhance human health. Ensuring the responsible development of these infrastructures guarantees the versatility and depth needed to address complex biomedical challenges in an ever-evolving landscape.
